# iPSC-Derived Cardiomyocytes as a Disease Model to Understand the Biology of Congenital Heart Defects

**DOI:** 10.3390/cells13171430

**Published:** 2024-08-26

**Authors:** Chithra K. Pushpan, Subramanyan Ram Kumar

**Affiliations:** 1 Division of Cardiothoracic Surgery, Department of Surgery, University of Nebraska Medical Center, Omaha, NE 68198-7616, USA; cpushpan@unmc.edu; 2 Dr. C.C. and Mabel, L. Criss Heart Center, Children’s Nebraska, 8200 Dodge St, Omaha, NE 68114, USA

**Keywords:** iPSC, cardiomyocyte, disease model, congenital heart diseases, TOF

## Abstract

The discovery of human pluripotent stem cells (hiPSCs) and advances in DNA editing techniques have opened opportunities for personalized cell-based therapies for a wide spectrum of diseases. It has gained importance as a valuable tool to investigate genetic and functional variations in congenital heart defects (CHDs), enabling the customization of treatment strategies. The ability to understand the disease process specific to the individual patient of interest provides this technology with a significant advantage over generic animal models. However, its utility as a disease-in-a-dish model requires identifying effective and efficient differentiation protocols that accurately reproduce disease traits. Currently, iPSC-related research relies heavily on the quality of cells and the properties of the differentiation technique In this review, we discuss the utility of iPSCs in bench CHD research, the molecular pathways involved in the differentiation of cardiomyocytes, and their applications in CHD disease modeling, therapeutics, and drug application.

## 1. Introduction

Recent advances in biomedical research ensure promising opportunities to benefit the overall well-being of human health by enabling preventive strategies, accurate diagnosis, and treatment options for each patient based on their unique characteristics. Human pluripotent stem cell-derived cardiomyocytes have emerged as a new platform to study cardiac diseases and regeneration by mimicking human disorders and the genetic mutations associated with them [1,2,3]. This was a breakthrough in regenerative medicine, disease modeling, and personalized drug discovery. This also offered an advantage in cardiovascular research as it could bypass the ambiguities that existed while converting data obtained from studying different model systems from different species with variable genetic characteristics. It is now possible to execute drug screenings using the specific patient sample as a disease-in-a-dish and study efficacy and toxicities. This approach is becoming very popular since it has a human origin, expanded and differentiated to the cell of interest, and the potential to generate personalized medicine [4,5].

Pluripotent stem cells were first generated from mouse fibroblasts and later from human fibroblasts. Initial findings in 2006 by Shinya Yamanaka’s group showed that iPSCs can be generated from mouse embryonic fibroblasts by overexpressing OCT4, Klf4, Sox2, and c-Myc using a gamma retroviral vector system [6]. These four factors are called Yamanaka factors. Subsequently, many different methods and factors have been studied and established to reprogram human somatic cells to iPSCs. Yu et al. studied other factors, Nanog and Lin28, in addition to OCT4 and Sox2 in human fibroblasts using a lentiviral vector [7]. Following this, several other somatic cells from various tissues were used to produce hiPSCs. Hanna et al. [8] proved that terminally differentiated B lymphocytes can be reprogrammed to pluripotent cells by using a few additional steps, including expression of C/EBPa and knockdown of the B cell transcription factor Pax. Linking another nuclear factor, an orphan nuclear receptor (Essrb), with Oct4 and Sox2 was demonstrated to reprogram mouse embryonic fibroblasts into iPSCs, which exhibited similar epigenetic features and gene expression as embryonic stem cells [8], but without exogenous Klf transcription factors. In 2011, Jai-Hee Moon et al. [9] reported that Bmi1, a protein involved in cell regulation, can reprogram mouse fibroblasts into iPSCs in combination with only Oct4, without Sox2, Klf4, and c-Myc. They also showed that activation of sonic hedgehog signaling using compounds such as Shh, purmorphamine, or oxysterol can be used instead of Bmi1 in combination with Oct4 to reprogram mouse fibroblasts into iPSCs [9]. Similarly, increased efficiency in reprogramming was observed with SV40 LT(T) when NANOG, KLF4 [10], LIN28, or MYC was omitted [11]. Valproic acid, BayK8644, and BIX-01294 [12], along with several other factors and chemical combinations, were successful in generating iPSCs from somatic cells. Factors including C/EBPa, p53 siRNA, UTF1, DNMT shRNA, Wnt3a, and hTERT and chemicals such as RG108, AZA, dexamethasone, sodium butyrate, TSA, SAHA, PD025901 + CHIR99021(2i), and A-83–01, as well as the induction of hypoxic conditions and the Mbd3 and p53 pathways, have also been described to enhance the efficiency of iPSC generation [13]. PBMCs are most commonly reprogrammed to generate iPSCs since they are easier to obtain, safer, and more economical [4]. There are two methods to induce iPSCs, integrative and non-integrative, based on whether they involve the incorporation of exogenous genetic material or genetic modification of the donor cells [13,14]. Retroviruses, lentiviruses, and plasma vectors are used under the integrative approach, and episomal DNA vectors, recombinant proteins, minicircle DNA, adenovirus, Sendai virus, synthetic mRNAs, etc., are used under the non-integrative approach. Chemical and environmental factors have also been shown to have a strong influence on accelerating reprogramming. 

Development of methodologies for differentiation of iPSCs into cardiomyocytes is achieved by utilizing the knowledge of signaling pathways involved in cardiac development, such as Activin/Nodal/TGF-beta, WNT, and BMP. Currently, lineage-specific development is achieved through the modulation of retinoic acid and Wnt signaling. Several combinations of growth factors and small molecules have been shown to enhance protocol efficiency and reproducibility [15]. This involves attaining a pure population of cardiomyocytes from a mixed population of cells using various methods such as cell surface markers, fluorescent probes, glucose deprivation, etc. Current efforts are aimed at the possibility of directing differentiation to a specific cardiomyocyte subtype rather than a heterogeneous pool of nodal, atrial, and ventricular myocytes, all of which can have different properties at molecular and functional levels. 

## 2. Differential Expression of Genes Drives Generation of Cardiomyocytes

**Figure 1:**
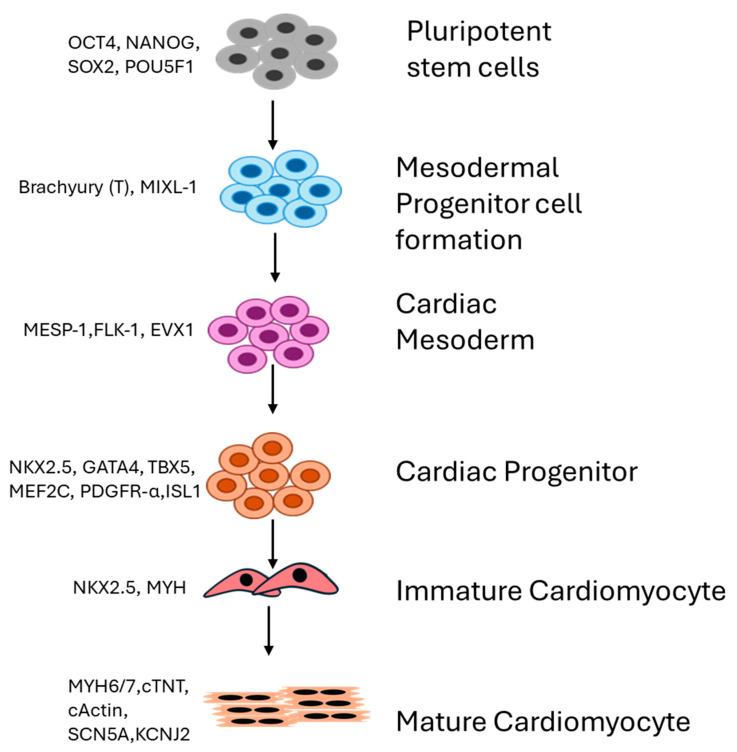
Schematic representation of different stages of cardiomyocyte differentiation from iPSCs. First stage is the formation of a primitive mesodermal streak followed by the cardiac mesoderm, cardiac progenitor cells, and differentiation into cardiomyocytes. Markers and transcription factors expressed at each stage are also shown.

**Figure 2:**
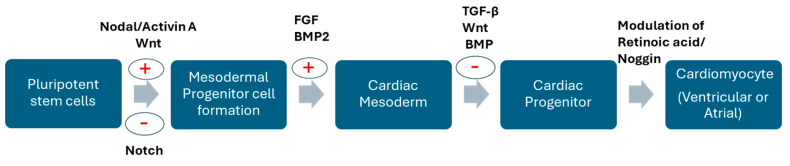
Signaling pathways involved at various stages in the differentiation of cardiomyocytes. hPSC-derived cardiomyocyte differentiation involves sequential exposure to various factors to transition to different stages. The Figure shows various signaling molecules involved in the transition of pluripotent stem cells to cardiomyocytes. FGF—fibroblast growth factor; BMP—bone morphogenetic protein; TGFβ—Transforming growth factor beta.

The differentiation of iPSCs into cardiomyocytes requires a series of orchestrated expressions of distinct sets of genes at each stage that mimic patterns seen in normal cardiomyocyte development. They can be distinguished into different stages—generation of the mesoderm, cardiogenic mesoderm, cardiac progenitors, and differentiated cardiomyocytes (Figure 1). Each stage requires the expression of specific genes as signals to drive cardiomyocyte formation. BMP4 or Bone morphogenetic protein 4 is a member of the TGF-beta superfamily that plays an indispensable role in cardiac mesoderm formation [16]. The extra-embryonic mesoderm secretes BMPs, creating a gradient that directs the differentiation of progenitor cells into cardiac mesoderm in spatial-, time-, and concentration-dependent manners. Complete deletion of BMP4 in embryos was shown to be lethal in mice [17,18], and conditional deletion has contributed to abnormal cardiac morphogenesis (causing right ventricle and outflow tract hypoplasia), demonstrating its role in the differentiation of second heart field (SHF) progenitors [19,20,21,22]. In addition to BMP4, the activin and nodal pathway (Figure 2) also plays important regulatory roles in the generation of cardiac mesoderm and is shown to be critical in atrioventricular canal-localized ECM organization. In zebrafish, it was shown that activin helps with epicardial progenitor migration to the developing heart tube [23]. The first step in cardiomyocyte development involves an environment with a low concentration of BMP4 and a high concentration of activin A-induced alpha-myosin heavy chains (Myh6) and FGF2 to induce primitive streak-like cells and mesoderm-expressing T, Wnt3a, and MIXL1. Then, Dickkopf (Dkk)1, VEGF, and FGF2 induce a shift to the next stage, which is the formation of cardiac mesoderm, characterized by the expression of *MESP1*, *ISL1*, and *KDR*. This step then induces KDRlow/C-KIT-neg multipotent progenitor cells that generate endothelial cells, smooth muscle cells, and cardiomyocytes. However, there are some reports showing dichotomies in the synergistic actions of BMP and FGFs in inducing progenitor cells. ActivinA with FGF2 [24], BMP, and VEGF induces the formation of progenitor cells committed to mesoderm formation and signaling cardiomyocyte, SMC, and endothelial cell formation [24]. Another important signaling pathway in developmental events is Wnt/beta-catenin signaling. The Wnt signaling pathway has a biphasic nature. In the early stages, it enhances the formation of the endoderm and mesoderm but inhibits the generation of cardiomyocytes in established mesoderm. Inhibition of canonical Wnt signaling by sFRP or the frizzled-related protein and Dickkopf1 (Dkk1), which are released from the endoderm, induces cardiac specification in the mesoderm after gastrulation [22,23,24,25]. Canonical Wnt signaling is also involved in the proliferation and maintenance of SHF progenitors and is specifically involved in the regulation of SHF [26,27], such that deletion or inhibition results in right ventricular and outflow tract hypoplasia in mice [26]. Another important factor that plays a role in the development of right ventricle and outflow tract (OFT) formation, chamber specification, trabeculation, and vascular SM development is Notch signaling [28]. Notch signaling interferes with Canonical Wnt signaling in SHF, inhibiting SHF progenitor proliferation and promoting differentiation [29,30]. Additionally, the hedgehog pathway (involved in the coronary vasculature, atrial septation, and OFT morphogenesis) and the Retinoic acid pathway (SHF derivative patterning and OFT and Atrial specification) [29,31] have been shown to accelerate cardiomyogenesis. Terminally differentiated cardiac cells express structural genes that regulate sarcomere-related proteins such as MYL2, MYL7, MYL6, and TNNT2). Although a high purity of cardiomyocytes can be generated using existing protocols, the major drawback is their immature state. A series of multiple complex protocols has to be applied to obtain a postnatal state. Mechanical stimuli, genetic regulation using chemical factors, 3D structures, etc., have been used to enhance the generation of adult-like cardiomyocytes [32]. Hsueh et al., 2023 demonstrated that the replacement of Wnt inhibitors by Sfrp2 can induce mature iPSC-derived cardiomyocytes. Three-dimensional systems such as engineered tissues and 3D organoids have been demonstrated as the most promising approach to attain maturation in vitro [33,34]. Reports show that co-culturing hPSC-CMs with fibroblasts and endothelial cells can improve iPSC-derived cardiomyocyte maturation as it helps to regulate cell morphology and stiffness and recapitulate the internal crosstalk normally seen in the myocardium [34,35,36]. Enhanced Ca^2+^ transient amplitudes, contraction rate, and contractility were observed in iPSC-derived CMs and hESC-derived CMs in combination with human primary cardiac microvascular endothelial cells and fibroblasts, suggesting a more mature contractile phenotype than CM microtissues [34,35,36]. Coculturing adult cardiac nonmyocytes with iPSC-CMs recapitulates the in vivo physiological environment and enhances maturation. The formation of the extracellular matrix and paracrine factors by cardiac fibroblasts, the formation of the microvasculature, and the regulation of metabolism and contractile function mediated by endothelial cells play important roles in the maturation of cardiomyocytes. Therefore, utilizing these cells in culture and for the maturation of iPSC-CMs can contribute to the generation of cardiomyocytes with a reliable phenotype [34,35,36].

Another major approach is by utilizing changes in myocardial metabolism during the fetal and adult stages. As opposed to the fetal stages, there are increased levels of lipid and oxygen concentrations in the blood of adults and this plays an important role in metabolic reprogramming. Adult cardiomyocytes utilize 80% of the energy from the beta-oxidation of fatty acids, whereas this figure is only 15% in fetal cardiomyocytes. Therefore, using fatty acid treatments and molecules that increase the expression of FA metabolism-related genes and markers for mitochondrial activity has been tested successfully to enhance cardiomyocyte maturation [34,37,38]. Therefore, a good understanding of maturation strategies is essential to obtain the optimal state required for individual studies. 

## 3. iPSC-Derived Cardiomyocytes in Disease Modeling

iPSC-CMs have recently been used as an important tool to study the pathophysiology of cardiac disorders. The most exciting application is the ability to generate patient-specific disease models in vitro. The modeling of human cardiac disorders using iPSCs offers immense opportunities to study the functional and molecular changes occurring in a specific disease process and the effect of various treatments in a patient-specific manner. This technology has enabled the development of more reliable in vitro models for very complex cardiac diseases. Patient cells can be collected using relatively less invasive approaches and reprogrammed to the cells of interest specific to the disease of interest. Additional advantages compared to existing methods include the extensive proliferative capacity of iPSCs, generation of cells from the exact patient of interest, thereby maintaining the mechanical and electrophysiological properties and genetic background, and avoidance of ethical constraints surrounding the use of embryonic stem cells. Cardiac diseases modeled using iPSC technology include arrhythmogenic right ventricular cardiomyopathy/dysplasia, long QT syndrome, left ventricular non-compaction, dilated cardiomyopathy, Timothy syndrome, Brugada syndrome, Andersen–Tawil syndrome, Leopard syndrome, and catecholaminergic polymorphic ventricular tachycardia, as well as metabolic disorders such as Friedreich’s ataxia, Barth syndrome, fatty acid oxidation disorders, and Pompe disease [39].

The electrophysiological characteristics of human cardiomyocytes are unique [15]. Therefore, establishing a human-cardiomyocyte-based disease model for understanding the pathophysiology of the disease process and for more efficient drug screening is crucial. Disease modeling using iPSC-derived CMs allows us to study the disease in a dish. The iPSC-driven disease-in-dish model can help in elucidating the molecular mechanisms and functional characteristics of cardiomyocytes unique to the diseased individual. This could enable the identification of new molecular targets and the design of personalized treatments based on the individual’s genetic makeup. CRISPR/Cas9-mediated gene editing of diseased iPSCs and transplantation to damaged heart tissue are strategies that could support cell-based therapies. In the future, it will be interesting to apply iPSC-driven disease-in-dish models to regenerative medicine, enabling the development of customized therapeutic strategies for individual patients.

Several different genetic and environmental factors are associated with the etiology of congenital heart diseases. Development of a normal, fully functional heart involves several spatiotemporally regulated signaling pathways, and any disruption in these pathways has the potential to cause a severe abnormality leading to CHDs. It is therefore important to understand which genetic mutation and variant is potentially associated with frequently observed CHDs. Some of the most studied CHDs are ventricular septal defects (VSDs), Tetralogy of Fallot (TOF), single ventricle defects (SVDs), Hypoplastic left heart syndrome (HLHS), Pulmonary Atresia With Ventricular Septal defects (PA-VSDs), double outlet right ventricle (DORV), Atrioventricular septal defects (AVSs), Patent ductus arteriosus (PDA), etc. [25,40,41,42,43,44,45]. Specifically, researchers have gained more insights into the underlying molecular mechanisms and studied transcriptional alterations in TOF and HLHS using iPSCs (Table 1). From these studies, it is now possible to understand the genetic makeup of some of these patients and develop potential therapeutic strategies. In the following sections, we will discuss the research on these abnormalities in more detail [40]. 

### 3.1. hiPSCs in Tetrology of Fallot

Tetrology of Fallot (TOF) is a cyanotic congenital heart defect which was first described in 1883 by pathologist Etienne-Louis Artur Fallot. It represents 5–7% of all congenital heart defects [46]. TOF is characterized by four structural abnormalities: right ventricular outflow tract obstruction, ventricular septal defect, overriding aorta, and right ventricular hypertrophy. Narrowing of the right outflow tract restricts blood flow to the lungs. There is a defect in the septum between the right and left ventricles, causing the mixing of deoxygenated and oxygenated blood. The aorta is displaced to the right side, positioning it to directly override the VSD, causing it to collect blood from both left and right ventricles and resulting in cyanosis due to the pumping of oxygen-poor blood into the circulation. There is hypertrophy of the right ventricle, whereby the right ventricle becomes thickened due to obstruction of the right ventricular outflow tract. The severity of the obstruction is clinically manifested as a cyanotic neonate. Usually, surgery is performed a few months after birth, where the VSD is closed and stenosis of the right outflow tract is relieved. The reported survival rate to the fifth decade of life is about 72% [14]. TOF is a conotruncal malformation that is caused by aberrant signaling during the early stages of embryogenesis, leading to defective development of the outflow tract of the heart. Loss of function or the mutation of several genes of the NOTCH, FLT4, and TBX1 transcription factors, which are involved in cardiac development, is seen in around 7% of patients affected with TOF [47]. NOTCH is a highly conserved cell signaling pathway involved in cell development, cell proliferation, death, and regeneration. It has been shown that NOTCH is critical for cardiac development and is involved in cardiac fate specification and patterning of the heart tube and cardiac structures [30]. FLT4 genes code for VEGF-C, which is involved in lymphatic vessel development. Mutations and variants of these genes are found in patients with TOF. In addition, TBX1, RYR1, ZFPMI, CAMTA2, DLX6, PCM1, NKX2.5, GATA-6, GATA-4, HAND1, HAND2, ZFPM2, NF-ATC, etc., which are involved in cardiac development, are labeled as potential candidates associated with TOF [48,49,50,51]. This shows that a wide range of gene mutations and variations can lead to TOF and an understanding of these variants in individuals can help unravel the genetic basis and similar conditions.

Use of hiPSC-derived cardiomyocytes as a disease model is powerful in studying the mechanism of disease from the early stages of development specific to the genetic abnormality in the given patient. For example, in a remarkable study by Paige et al., 2019, it was shown in a large cohort of 829 TOF patients that deleterious mutations affected cell growth and the development of the heart using whole-genome sequencing. This study showed that NOTCH1 harbored unique and deleterious variants. NOTCH1 variants also showed a reduction in Jagged1-induced NOTCH signaling. A total of 22 unique deleterious variants were found in FLT4 variants in TOF patients. These results suggested that there are significant alterations in cell proliferation and migration; however, the role of these genetic changes in the early stages of cardiogenesis was not completely understood due to a lack of in vitro models. However, with the advances in iPSC technology, this limitation could be overcome. Grunert et al. used patient-specific iPSCs from TOF patients to analyze gene expression patterns and variability using whole-genome and transcriptome sequencing data in 2020 [40,48,49,50]. They examined cardiomyocytes derived from iPSC lines from two well-characterized patients and compared them with control iPSCs from healthy individuals. They demonstrated that two out of three clones of a TOF sample (TOF-02) had a somatic mutation in the DNA-binding domain of tumor suppressor P53, which was not seen in blood genomic DNA. Differences in gene expression in various stages of cardiac differentiation were observed when compared to controls, providing the first investigatory report of transcriptional alterations and molecular mechanisms leading to TOF. Besides mutations analyzed by whole-genome sequencing and transcriptome analysis, they observed that there was an increased number of differentially expressed genes during differentiation between TOF patients and healthy individuals for derived cardiomyocytes on different days (day 15 vs. day 60). Collagen-related genes and some disease-related genes such as BICC1 and MYH11 were differentially expressed in iPSC-CMs from TOF, both of which have distinct and crucial roles in cardiovascular health. MYH11 is involved in smooth muscle contraction and variants in MYH11 are associated with thoracic aneurysm and patent ductus arteriosus. BICC1 is an RNA-binding protein involved in the regulation of gene expression and acts as a negative regulator of the Wnt signaling pathway during embryonic development. It is implicated as a potential marker for cardiorenal syndrome and heart failure [52,53]. The observed differences in genes related to Wnt signaling were demonstrated throughout different stages of maturation for patients and healthy controls. A disease-causing mutation was also observed in FBLN2, a protein involved in the VEGFA pathway [40], as well as NOTCH signaling and DAAM2, required for sarcomere assembly and myocardial maturation in iPSC-derived CMs from TOF patients, suggesting that VEGF downregulation can enhance the risk for TOF. Overall, the study showed that there are significant differences in gene expressions in iPSC-CMs from patients and healthy individuals, providing a primary investigation using iPSCs to effectively elucidate the underlying mechanisms of TOF.

Kitani et al. recently published an RNA sequencing analysis of iPSC-CMs from TOF patients, SVD (single ventricle disease) patients, and healthy individuals. Gene expression patterns showed an increase in differentially expressed genes in SVD-iPSC-CMs and TOF-iPSC-CMs. The enriched DEGs were related to developmental processes. A transcription factor binding analysis identified several TF binding motifs enriched in CHD-iPSC-CMs, such as IRF, TGIF, and NF-κB. In SVD-iPSC-CMs, there was greater enrichment in DEG and transcriptional factor binding motifs but there was only a modest increase in TOF-iPSCs. They also showed that the SVD and TOF iPSC-CMs express distinctive transcriptomes when compared with those from healthy individuals. More research using iPSC-derived CMs from patients can help us to understand the significance of the findings with relevance to disease progression [41]. 

### 3.2. hiPSCs in Hypoplastic Left Heart Syndrome

Hypoplastic left heart syndrome (HLHS) is a severe form of congenital heart disease characterized by the underdevelopment of structures in the left side of the heart, which include hypoplasia of the left ventricle and hypoplasia or atresia of the mitral valve, aortic valve, and ascending aorta. It is a life-threatening condition that requires surgical intervention soon after birth [54]. Jiang et al., 2014 [43], used iPSCs from HLHS patients who died 10 days after birth to derive CMs, comparing them with hESCs and control iPSCs to evaluate the differences in genetic factors that contribute to the development of the HLHS phenotype. They report an impairment in the differentiation process to the cardiac lineage of fully formed mature CMs. They observed a reduction in CX43 expression in iPSC-derived CMs from HLHS patients, showing that there is impaired electromechanical transduction which causes the failure of correct cardiac myocyte alignment. CX40, which is related to the ventricular conduction system, was higher and correlated to an increased number of VCS in HLHS. HLHS iPSC-CMs had an irregular myofibril arrangement, RER, and SR. The iPSC-CMs showed lower beating rates and accelerated Ca^2+^ transient decay. Ryanodynie receptor dysfunction and sarcoplasmic reticulum dysfunction were confirmed by varying responses to calcium transients and on treatment with the β1/β2 adrenergic receptor agonist isoproterenol and electrophysiological responses measured by a microelectrode array. These results suggest an immature CM differentiation process in patients with HLHS.

Kobayashi et al. showed that there is a decreased potential for cardiomyogenic differentiation, repressed transcription of NKX2–5, decreased levels of TBX2 and NOTCH/HEY, and inhibited HAND1/2 signaling in HLHS. They demonstrated reduced H3K27 dimethylation but increased H3K27 trimethylation, causing inhibition of the transcriptional activation of the NKX2–5 promoter. Their approach suggests that iPSC-CMs may be used to elucidate epigenetic changes and complex transcriptional mechanisms that underlie HLHS [55,56]. In 2020, Paige et al. reported impaired contractility in HLHS using patient-specific iPSC-CMs. They explored the mechanisms that caused the pathogenesis of right ventricle failure despite left ventricle-specific hypoplasia in the HLHS patient population. They used iPSC-CMs from HLHS patients who developed RV failure and control lines from healthy individuals. They observed a decrease in contraction force acceleration in HLHS iPSC-CMs but no disorganization in the sarcomere structure. Analysis of differentially expressed genes from G1-phase iPSC-CMs from HLHS patients using single-cell RNA sequencing revealed an increased expression of cytoskeletal and sarcomere genes but a decrease in the expression of genes related to metabolism and mitochondrial function. There is a reduction in mitochondrial content in HLHS-iPSC-CMs in comparison to control iPSC-CMs, which could contribute to impaired contractility. Their experiments also suggest that altering the energy source or substrate can reverse the contractile variability and conclude that RV failure in HLHS may be due to this functional impairment. These findings open new avenues for therapeutic targets [55].

### 3.3. hiPSCs in Pulmonary Atresia with Intact Ventricular Septum

Pulmonary atresia with intact ventricular septum (PA-IVS) is a rare defect where structures of the right side of the heart are underdeveloped to varying degrees, resulting in variable amounts of antegrade pulmonary blood flow. Little information about the underlying etiologic factors exists and no animal models with this phenotype have been established yet. Patient samples from biopsies serve as the sole source of PAIVS-CMs for studying genetic alterations. Although the studies identified mutations in mature cardiomyocytes, the alterations during the fetal stages and development remain unclear. iPSC-CMs from PA-IVS patients were used to study intrinsic functional properties and molecular changes in cardiac development using single-cell transcriptomes and constructed bioengineered tissues [57]. Using bioengineered tissues, the authors demonstrate that contractility is impaired. scRNA-seq showed downregulation of genes comprising the contractile apparatus and cardiac maturation but an increase in the expression of immature isoforms. They explained this as the underlying cause for persistent RV systolic and diastolic dysfunction even after completing biventricular repair in PA-IVS. This was the first study that used iPSCs to model PA-IVS. Yu et al., 2024 used iPSC-CMs from PA-IVS patients with single ventricle palliation and biventricular repair to study the underlying molecular mechanism of the pathology of right ventricular hypoplasia. They showed that there is increased mitochondrial respiration, decreased cardiomyocyte proliferation, and enhanced maturation. They also observed that there is impaired differentiation towards SHF progenitors and enhanced differentiation towards FHF and epicardial lineages in PA-IVS with single ventricle palliation [54].

### 3.4. hiPSC in Drug Screening

Long QT syndrome (LQTS) is one of the conditions examined using iPSCs [58]. LQTS is a life-threatening cardiovascular condition that causes malignant arrhythmia characterized by a prolonged QT interval on ECGs, increasing the time between heartbeats [59,60]. Patients with LQTS undergo syncopal episodes, abnormalities on ECGs, and palpitations, which may lead to sudden death. Congenital LQTS accounts for 75% of all LQTS cases [59,61]. LQTS can be inherited and acquired. There are different subtypes of LQTS and it is caused by a genetic disorder with an autosomal dominant inheritance trait and mutations in the KCNQ1, KCNH2, and SCN5A genes [62,63,64]. LQTS1, LQTS2, LQTS3, LQTS7, LQTS8, and LQTS14/15 have been studied using iPSCs [39,65]. LQTS1 has mutations in the KCNQ1 gene, while LQT2 and LQT3 have mutations in the KCNH2 gene and SCN5A gene, respectively. Moretti et al., 2010 [66] used iPSC-CMs derived from two family members carrying a missense mutation in the KCNQ1 gene that encodes the ion channel responsible for adrenergic-sensitive, slow outward potassium currents I(Ks). The hiPSC-derived CMs showed a significant decrease in I(ks) current and alterations in channel activation and deactivation when compared to normal healthy individuals. Moreover, the beta-blockade of LQT1 iPSC-CMs showed a protective role against catecholamine-induced tachyarrhythmia.

In a recent study by Wang et al. 2022., they employed hiPSC technology to reprogram somatic cells from LQTS patients into iPSCs to generate a personalized disease modeling platform [67]. Sequencing data determined that the patient is carrying not only a heterozygous KCNQ1 c.656G > A mutation but also a heterozygous TRPM4 c.479C > T mutation. It was interesting to see that there was a unique dual mutation combination, which might have contributed to the ineffectiveness of mainstream LQTS medications. 

Similarly, the use of patient-derived iPSCs was shown to recapitulate disease phenotype by several groups and has been demonstrated to be a useful tool to accurately study the patient-specific mutations and variability in drug responses. Egashira et al., 2012 had previously studied drug responses in iPSC-derived CMs from a sporadic LQTS patient with a novel heterozygous mutation in the KCNQ1 gene, 1893delC. They screened several drugs and confirmed the channel responsible for the disease phenotype. They studied electrophysiological properties using MEA plates. In their study with iPSC-derived cardiomyocytes, the effect of administering an I(Ks) blocker on cFPD in LQTS was investigated and compared to control samples from healthy adults. However, the IKr blocker prolonged cFPD in LQTS and the control. EAD- and PVT-like arrhythmias were recorded for LQTS alone. They observed that cFPD was not affected in LQTS but prolonged in controls. Taken together, they proposed that I(Ks) channels were impaired and demonstrated that *KCNQ1* 1893delC has a dominant negative effect via a trafficking deficiency [68]. This shows that disease phenotypes can be modeled using hiPSC-derived cardiomyocytes, providing a valuable tool that can be used to screen drugs and identify potential targets for diseases in a patient-specific manner.

## 4. Limitations of iPSCs as a Model System

The challenges involved in the use of iPSCs are primarily issues with reprogramming efficiency, ideal differentiation protocols, and the differentiation capabilities of the cells. There are concerns regarding the maturation of iPSC-derived cardiomyocytes, the intrinsic differences between adult CMs and hiPSC-CMs, and the generation of potential variability. There is a very low efficiency of conversion to iPSCs and genomic integration, which can contribute to potential risk. Spontaneous mutations in clones and the possibility of off-target gene modifications, although very rare, have been described. Currently, gene editing tools are expensive but with advancements in technology, a fall in price can be anticipated [69,70].

## 5. Conclusions

Advances in iPSC technology have immense potential to contribute to regenerative medicine. The possibility of using patient-derived iPSCs to recapitulate disease phenotype makes it a useful tool to accurately study patient-specific mutations. This enables researchers to understand the underlying molecular mechanism causing the diseases, making genotype-to-phenotype correlations possible, thereby bridging the gap between basic research and clinical applications. The ability to generate three-dimensional cardiac organoids [71] that mimic the complex aspects of the heart provides promising opportunities in translational research. The use of iPSC-CMs in drug screening or drug toxicity can provide information on variability in drug responses, extending their potential to the development of personalized medicine and therapy. 

**Table 1:** Different Strategies Employed for using iPSCs to Study Underlying Mechanisms in CHDs.

Condition	Disease Model	Experimental Methods	Observation	Reference
TOF	Patient-specific TOF-iPSC-derived CMs	Whole genome and transcriptome sequencing of iPSC-CMs from TOF patients and healthy individuals	(1) Somatic mutation in DNA-binding domain of P53 in one clone—May point to functional impact, (2) Increased DEG observed in control vs. TOF patient CMs, (3) Collagen-related genes are more affected, (4) BICC1 and MYH11 downregulated in CM d60 in TOF and mutation in FBLN2 and DAAM2	Grunert et al., 2020 [40]
TOF and SVD	Patient-specific TOF and SVD-iPSC-derived CMs	RNA Sequencing Analysis of iPSC-CM from TOF, SVD, and healthy individuals	Significantly distinct transcriptomes in iPSC-CMs from SVD and TOF compared to non-CHD iPSC-CMs	Kitani et al., 2020 [41]
TOF and SVD	Patient-specific TOF and SVD-iPSC-derived CMs	RNA-seq datasets of Patient-specific TOF and SVD-iPSC-derived CMs from Gene Expression Omnibus database used to study genome-wide Alternative splicing (AS) events	Abnormal AS events observed in TOF and SVD iPSC-CMs compared to non-CHD iPSC-CMs	Xu et al., 2022 [42]
HLHS	Patient-specific HLHS-iPSC-derived CMs	Investigated cellular, structural, and functional properties of HLHS iPSC-CMs. Employed RNA-seq analysis and whole-exome sequencing analysis	Decreased beating rate, disorganised sarcomeres, dysfunctional sarcoplasmic reticulum, and deleterious variants in NOTCH receptor and genes involved in its signaling pathway	Yang et al., 2017 [72]
HLHS	Patient-specific HLHS-iPSC-derived CMs	iPSC-CMs from HLH patients, sarcomere immunostaining. Examined the impact of MYH6-R443P variant by inserting it into iPSCs from healthy individuals	Insertion of variant caused dysmorphic sarcomere phenotype and contractile dysfunction in healthy iPSC-CMs. Correcting the MYH6-R443P variant using CRISPR/Cas9 gene editing rescued the iPSC-CMs from reduced efficiency in CM differentiation, sacromere dysmorphism, and abnormal contraction rates	Kim et al., 2020 [44]
HLHS	Patient-specific HLHS-iPSC-derived CMs	Comprehensive analysis of HLHS-iPSC-CMs for testing the efficiency of cardiomyocyte differentiation, contractility and calcium handling, mPTP closure, mitochondrial dynamics, and respiration and corroborated the results with scRNA-seq analysis	Mitochondrial dysfunction and oxidative stress in HLHS; points to potential therapeutic interventions by targeting mPTP closure with sildenafil or suppression of UPR with TUDCA	Xu et al., 2022 [45]
HLHS	Patient-specific HLHS-iPSC-derived endocardial endothelial cells co-cultured with iPSC-CMs	Single-cell transcriptomic analysis of HLHS-iPSC endocardium and human fetal heart tissue	Defect in endocardial function and endocardium–myocardium cross-talk in HLHS	Miao et al., 2020 [73]
PA-IVS-1v	Patient-specific PA-IVS-1v-iPSC-CMs	Single-cell RNA sequencing and assessment of biological pathways and metabolic functions	Proportion of SHF progenitors reduced, FHF and epicardial progenitors increased. Seahorse assays show enhanced mitochondrial activity. Enhanced ATP and respiration in PA-IVS-1v compared to control and cardiac proliferation is compromised	Yu et al., 2024 [54]
PA-IVS	Patient-specific PA-IVS-iPSC-CMs	Single-cell transcriptomics and functional analysis of PA-IVS-iPSC-CMs and their engineered tissues models	Showed a downregulation of RV developmental pathways, contractility, and cardiac maturation	Lam et al., 2020 [57]
